# Group A *Streptococcus* Meningitis, United States, 1997–2022

**DOI:** 10.3201/eid3201.250871

**Published:** 2026-01

**Authors:** Paulina A. Hawkins, Sopio Chochua, Namrata Prasad, Jennifer O. Okaro, Yuan Li, Tasha Martin, Ann Thomas, Bridget J. Anderson, Kari E. Burzlaff, Lee Harrison, Shannon Seopaul, Nisha Alden, Rachel Herlihy, William Schaffner, H. Keipp Talbot, Ruth Lynfield, Kathy Como-Sabetti, Maria Rosales, Shua Chai, Sam Sefton, Jessica R. Howard-Anderson, Sarah Khanlian, Jessica Houston, Susan Petit, Adam L. Cohen, Christopher J. Gregory

**Affiliations:** Centers for Disease Control and Prevention, Atlanta, Georgia, USA (P.A. Hawkins, S. Chochua, N. Prasad, J.O. Okaro, Y. Li, S. Chai, A.L. Cohen, C.J. Gregory); Oregon Health Authority, Portland, Oregon, USA (T. Martin, A. Thomas); New York State Department of Health, Albany, New York, USA (B.J. Anderson, K.E. Burzlaff); Bloomberg School of Public Health, Johns Hopkins University, Baltimore, Maryland, USA (L. Harrison, S. Seopaul); Colorado Department of Public Health and the Environment, Denver, Colorado, USA (N. Alden, R. Herlihy); Vanderbilt University School of Medicine, Nashville, Tennessee, USA (W. Schaffner, H.K. Talbot); Minnesota Department of Health, St. Paul, Minnesota, USA (R. Lynfield, K. Como-Sabetti); California Emerging Infections Program, Oakland, California, USA (M. Rosales, S. Chai); Emory University School of Medicine, Atlanta (S. Sefton, J.R. Howard-Anderson); New Mexico Department of Health, Santa Fe, New Mexico, USA (S. Khanlian, J. Houston); Connecticut Department of Public Health, Hartford, Connecticut, USA (S. Petit)

**Keywords:** *Streptococcus pyogenes*, meningitis/encephalitis, bacteria, bacterial infection, group A Streptococcus, streptococcal infection, United States

## Abstract

Group A *Streptococcus* (GAS) causes a variety of diseases in humans but is not widely appreciated as a cause of meningitis. During 1997–2022, ten sites participating in the Active Bacterial Core Surveillance network in the United States identified GAS meningitis cases. We calculated annual incidence and case-fatality rates (CFRs) for 320 of those cases and determined antimicrobial resistance by whole-genome sequencing. Annual incidence of GAS meningitis ranged from 0.02 to 0.07 cases/100,000 persons. Children <1 year of age had the highest average annual incidence, 0.23 cases/100,000 children. GAS meningitis had a higher CFR (19.4%) than meningitis caused by group B *Streptococcus*, *Streptococcus pneumoniae*, *Neisseria meningitidis*, or *Haemophilus influenzae*. Clindamycin resistance among GAS meningitis isolates increased from 3.2% during 1997–2002 to 17.7% during 2018–2022. Clinicians should be aware that meningitis is an uncommon but severe manifestation of invasive GAS and has a higher CFR than more established meningitis etiologies.

Group A *Streptococcus* (GAS) infections include invasive GAS (iGAS) infections, which are associated with high case-fatality rates (CFRs) (*1*). Clinical manifestations of iGAS disease include cellulitis, bacteremia, pneumonia, necrotizing fasciitis, and streptococcal toxic shock syndrome (STSS) (*1*,*2*). Treatment for iGAS infection includes supportive care and antimicrobial treatment, including combination therapy with penicillin and clindamycin in severe infections (*3*). GAS vaccine candidates in development include a 30-valent M protein–based vaccine (*4*).

Meningitis is an uncommonly reported but severe manifestation of iGAS infection (*1*). Among 91 pediatric intracranial GAS infections in the United States during 1997–2014, meningitis was the most common illness and was associated with the highest CFR (*5*). Meningitis was responsible for 2% of severe GAS disease cases in Europe during 2003–2004 (*6*), and GAS was responsible for 2% of community-acquired bacterial meningitis cases in the Netherlands during 2006–2013 (*7*). Analyses from several countries have identified recent increases in GAS meningitis, potentially associated with changes in strain characteristics (*8*,*9*). 

In contrast to other bacterial pathogens such as *Streptococcus pneumoniae* (pneumococcus), group B *Streptococcus* (GBS), *Haemophilus influenzae*, *Neisseria meningitidis* (meningococcus), and *Listeria monocytogenes* (*10*), GAS has not been widely recognized as a cause of meningitis. Thus, GAS infection has not been included in clinical practice guidelines for central nervous system infections (*11*) or recent reviews of bacterial meningitis (*12*). Trends in incidence rates and clinical and microbiological characteristics of GAS meningitis cases in the United States have not been as thoroughly described as other meningitis etiologies (*10*). We used active laboratory- and population-based GAS surveillance to describe incidence, demographic and clinical characteristics, and associated *emm* types and antimicrobial resistance profiles for GAS meningitis in the United States during 1997–2022. We compared those findings to other manifestations of GAS disease and other bacterial meningitis etiologies to more thoroughly describe epidemiology of GAS-related meningitis.

## Materials and Methods

### Surveillance

The Active Bacterial Core surveillance (ABCs) system (*13*), within the Emerging Infections Program Network of the Centers for Disease Control and Prevention, identifies GAS meningitis cases and other invasive bacterial infections. From 1997 to 2015, areas covered under the ABCs system expanded (Appendix Table). By 2022, ABCs areas included ≈34.9 million residents in 10 states.

ABCs defines iGAS as GAS isolated from a normally sterile site, or from a wound culture if accompanied by a diagnosis of necrotizing fasciitis or STSS, in a resident of a surveillance area (*13*). Consistent with previous ABCs reports (*13*), we defined a meningitis case as an ABCs pathogen (*S. pneumoniae*, GAS, GBS, *H. influenzae*, *N. meningitidis*) isolated from cerebrospinal fluid in any patient or from another sterile site in a patient with a clinical diagnosis of meningitis in the medical record (*8*). For this study, we included all ABCs cases identified during January 1, 1997–December 31, 2022.

### Laboratory Testing

We performed whole-genome sequencing (WGS) on all available isolates from GAS meningitis cases, as previously described (*14*,*15*). We analyzed sequences by using a previously validated bioinformatics pipeline to identify *emm* type and multilocus sequence type (ST), predict antimicrobial resistance, and determine the presence of select virulence factors (*14*,*15*).

Before 2015, we performed antimicrobial susceptibility testing by using broth microdilution, as previously described (*14*). Starting in 2015, we predicted susceptibility by WGS, on the basis of detection of resistance determinants (*14*,*15*), or by using a penicillin-binding protein 2× typing scheme for β-lactams (*14*). Those methods are comparable, as previously reported (*15*).

### Statistical Analyses

We calculated annual GAS meningitis incidence, expressed as the number of cases per 100,000 persons, stratified by age (<18 years and >18 years), by using US Census annual population estimates for ABCs catchment areas. We also calculated average annual incidence of meningitis caused by each ABCs pathogen, stratified by age group (0–11 months, 1–4 years, 5–17 years, 18–64 years, >65 years), during 3 time periods: before the meningococcal conjugate vaccine (MCV4) was introduced (1997–2005); after MCV4 was introduced, and during introduction of pneumococcal conjugate vaccine (PCV) 7, but before introduction the meningococcal B (MenB) vaccine (2006–2015); and after introduction of both meningococcal vaccines and 2 pneumococcal vaccines (PCV7, PCV13) (2016–2022). All time periods occurred after introduction of the *H. influenza* type b (Hib) vaccine. For that comparison, we limited catchment areas to the areas common to all ABCs pathogens. To estimate the national burden of GAS meningitis in the United States, we applied age- and race-specific observed incidence of GAS meningitis from the ABCs data to the total US population.

We calculated CFR on the basis of outcome at discharge. We compared categorical variables by using Fisher exact test or χ^2^ test and considered p<0.05 statistically significant. We examined linear trends by using linear regression and a *t*-test or by using the Cochran-Armitage test for binomial proportions.

We defined an isolate as covered by the 30-valent vaccine when its *emm* types were included in the vaccine, that is, *emm* types 1, 2, 3, 4, 5, 6, 11, 12, 14, 18, 19, 22, 24, 28, 29, 44, 49, 58, 73, 75, 77, 78, 81, 82, 83, 87, 89, 92, 114, and 118 (*4*). We calculated changes in the percentage of clindamycin-resistant isolates and distribution of *emm* types over 5 time periods of similar length: 1997–2002, 2003–2007, 2008–2012, 2013–2017, and 2018–2022.

## Results

### Demographic and Clinical Characteristics of GAS Meningitis Case-Patients

During 1997–2022, we identified a total of 38,262 cases of iGAS infection through ABCs, among which 320 (0.84%) met our meningitis definition. Most (69.3%) GAS meningitis cases occurred during December–May annually (Appendix Figure 1).

Of the 320 GAS meningitis cases, 112 (35.0%) occurred in children (persons <18 years of age) (Table 1). During the same timeframe, children accounted for only 8.8% (n = 3,342) of nonmeningitis iGAS cases (n = 37,935). The median age of patients with GAS meningitis was 41 (range 0–92, IQR 8–58) years, significantly lower than the median age of 53 (range 0–107, IQR 36–68) years among nonmeningitis iGAS patients (p<0.001). We saw no statistically significant difference in sex, race, or ethnicity between iGAS patients with and without meningitis (Tables 1, 2).

**Table 1:** Demographic and clinical characteristics of pediatric patients with group A *Streptococcus* meningitis versus other nonmeningitis invasive group A *Streptococcus* infections, United States, 1997–2022*

Characteristics	Meningitis, n = 320	Nonmeningitis, n = 37,942	p value
Pediatric patients	112 (35.0)	3,342 (8.8)	<0.001
Age range			
0–11 mo	24 (21.4)	471 (14.1)	**0.04**
1–4 y	25 (22.3)	1,134 (33.9)	**0.01**
5–17 y	63 (56.3)	1,736 (52.0)	0.37
Sex			
M	56 (50)	1,943 (58.1)	0.09
F	56 (50)	1,399 (41.9)	
Race and ethnicity			
White non-Hispanic	43 (38.4)	1,109 (33.2)	0.25
Black non-Hispanic	18 (16.1)	540 (16.2)	>0.99
American Indian/Alaska Native non-Hispanic	2 (1.8)	73 (2.2)	0.85
Asian/Pacific Islander non-Hispanic	5 (4.5)	188 (5.6)	0.64
Other non-Hispanic	0 (0.0)	7 (0.2)	0.24
Hispanic	24 (21.4)	664 (19.9)	0.67
Unknown	20 (17.9)	761 (22.7)	0.22
Underlying conditions			
Chronic medical conditions†	4 (3.6)	381 (11.4)	**0.004**
Immunocompromising conditions‡	1 (0.9)	90 (2.7)	0.25
Obesity	2 (1.8)	103 (3.1)	0.47
None	99 (88.4)	2,673 (80.0)	**0.004**
No data available	6 (5.4)	157 (4.5)	
Co-occurring syndromes			
Otitis media	6 (5.4)	NA	NA
Pneumonia	11 (9.8)	NA	NA
Abscess	7 (6.8)	NA	NA
Septic shock	9 (8.0)	NA	NA
Cellulitis	9 (8.0)	NA	NA
Streptococcal toxic shock syndrome	4 (3.6)	NA	NA
None of the above	78 (69.6)	NA	NA
Outcomes			
Died (CFR)	19 (17.0)	106 (3.2)	**<0.001**
Vaccine target coverage			
*emm* type data available	94 (83.9)	2,616 (78.3)	
*emm* type in 30-valent vaccine§	86 (91.5)	2,440 (93.3)	0.49
Antimicrobial susceptibility			
Susceptibility data available	91 (81.3)	2,079 (62.2)	
Nonsusceptible			
Erythromycin	4 (4.4)	163 (7.9)	0.23
Clindamycin	4 (4.4)	103 (4.9)	0.86
Levofloxacin	1 (1.1)	16 (0.8)	0.68

*Bold font indicates statistical significance. CFR, case-fatality rate; NA, not applicable †Chronic medical conditions included asthma, chronic obstructive pulmonary disease, diabetes, cirrhosis, alcohol abuse, atherosclerotic cardiovascular disease, congestive heart failure, burns, cerebrospinal fluid leak, and cerebrovascular accident. ‡Immunocompromising conditions included multiple myeloma, sickle cell disease, asplenia, organ transplantation, immunoglobulin deficiency, immunosuppressive therapy, human immunodeficiency virus or the acquired immunodeficiency syndrome (HIV–AIDS), leukemia, Hodgkin’s disease, lupus, nephrotic syndrome, and chronic kidney disease. §30-valent vaccine *emm* types include 1, 2, 3, 4, 5, 6, 11, 12, 14, 18, 19, 22, 24, 28, 29, 44, 49, 58, 73, 75, 77, 78, 81, 82, 83, 87, 89, 92, 114, and 118.

**Table 2:** Demographic and clinical characteristics of adult patients with group A *Streptococcus* meningitis versus other nonmeningitis invasive group A *Streptococcus* infections, United States, 1997–2022*

Characteristics	Meningitis, n = 320	Nonmeningitis, n = 37,942	p value
Adult patients	208 (65.0)	34,600 (91.2)	**<0.001**
Age range, y			
18–64	153 (73.6)	23,106 (66.7)	
>65	55 (26.4)	11,494 (33.2)	**0.04**
Sex			
M	93 (44.7)	19,193 (55.5)	**0.002**
F	115 (55.3)	15,407 (44.5)	
Race and ethnicity			
White non-Hispanic	102 (49.0)	15,432 (44.6)	0.20
Black non-Hispanic	24 (11.5)	3,943 (11.4)	0.93
American Indian/Alaska Native non-Hispanic	2 (0.9)	1,142 (0.3)	0.06
Asian/Pacific Islander non-Hispanic	3 (1.4)	702 (2.0)	0.59
Other non-Hispanic	0	12 (0.03)	0.07
Hispanic	18 (8.7)	3,495 (10.1)	0.50
Unknown	59 (28.4)	9,391 (27.2)	0.69
Underlying conditions			
Chronic medical conditions†	102 (49.0)	21,740 (62.8)	**<0.001**
Immunocompromising conditions‡	24 (11.5)	4,887 (14.1)	0.29
Obesity	50 (24.0)	9,565 (27.7)	0.25
Smoking	22 (12.9)	4,432 (16.5)	0.34
Experiencing homelessness	12 (9.8)	2,778 (12.0)	0.23
Living in longterm care facility	4 (3.3)	1,538 (6.7)	0.06
Intravenous drug use	12 (5.8)	4,225 (12.2)	**0.002**
None of the above	61 (29.3)	7,335 (21.2)	0.06
Unknown	5 (2.4)	589 (1.7)	
Co-occurring syndromes			
Otitis media	38 (18.3)	NA	NA
Pneumonia	27 (12.9)	NA	NA
Abscess	15 (8.0)	NA	NA
Septic shock	27 (13.0)	NA	NA
Cellulitis	18 (8.7)	NA	NA
Streptococcal toxic shock syndrome	6 (2.9)	NA	NA
None of the above	113 (52.9)	NA	NA
Outcome			
Died (CFR)	43 (20.7)	3,936 (11.4)	**<0.001**
Vaccine target coverage			
*emm* type data available	169 (81.3)	28,568 (82.6)	
*emm* type in 30-valent vaccine§	152 (89.9)	24,235 (84.8)	0.06
Antimicrobial susceptibility			
Susceptibility data available	167 (80.3)	25210 (72.9)	
Nonsusceptible			
Erythromycin	20 (11.9)	5,217 (20.7)	**0.004**
Clindamycin	17 (10.2)	4,571 (18.1)	**0.005**
Levofloxacin	1 (0.6)	267 (1.1)	0.64

*Bold font indicates statistical significance. CFR, case-fatality rate; NA, not applicable †Chronic medical conditions included asthma, chronic obstructive pulmonary disease, diabetes, cirrhosis, alcohol abuse, atherosclerotic cardiovascular disease, congestive heart failure, burns, cerebrospinal fluid leak, and cerebrovascular accident. ‡Immunocompromising conditions included multiple myeloma, sickle cell disease, asplenia, organ transplantation, immunoglobulin deficiency, immunosuppressive therapy, human immunodeficiency virus or the acquired immunodeficiency syndrome (HIV–AIDS), leukemia, Hodgkin’s disease, lupus, nephrotic syndrome, and chronic kidney disease. §30-valent vaccine *emm* types include 1, 2, 3, 4, 5, 6, 11, 12, 14, 18, 19, 22, 24, 28, 29, 44, 49, 58, 73, 75, 77, 78, 81, 82, 83, 87, 89, 92, 114, and 118.

Of 112 pediatric patients with GAS meningitis, only 6.2% had >1 underlying condition, compared with 15.3% of pediatric iGAS cases with nonmeningitis syndromes (p<0.01) (Table 1). Among adults (persons >18 years of age), 70.7% of GAS meningitis patients and 78.8% of nonmeningitis iGAS patients had >1 underlying condition (Table 2).

Across all age groups, 40.3% of patients with GAS meningitis had >1 additional iGAS clinical syndrome, such as otitis media, pneumonia, abscess, septic shock, cellulitis, or STSS, documented in ABCs. The most common clinical syndromes co-occurring with GAS meningitis were pneumonia (9.8%) among children and otitis media (18.3%) among adults (Tables 1, 2). The CFR in adults increased from 20.7% (43/208) to 51.6% (16/31) when meningitis was complicated by septic shock or STSS (p<0.01).

The overall CFR for GAS meningitis was 19.4%; CFR was 20.8% among persons <1 year of age, 24.0% among persons 1–4 years of age, 12.7% among persons 5–17 years of age, 20.9% among persons 18–64 years of age, and 20.0% among persons >65 years of age. We saw no statistically significant variation in CFR across the years of the study in any of the age groups. Overall, 4,104 iGAS case-patients died during the study period, and 62 (1.5%) of those deaths were caused by meningitis. Of the 125 deaths among pediatric iGAS cases, 19 (15.2%) were caused by meningitis.

### GAS Meningitis Incidence Trends and National Estimates

Annual GAS meningitis incidence fluctuated little across the study period, from 0.02 to 0.07 cases/100,000 persons, representing 0.3%–1.9% of iGAS infections in ABCs each year (Appendix Figure 2). In contrast, the incidence of all iGAS infections in ABCs remained stable during 1997–2013 (3.1 to 4.2 cases/100,000 persons), before beginning to rise in 2014, reaching 8.2 cases/100,000 persons in 2022.

**Figure 1:**
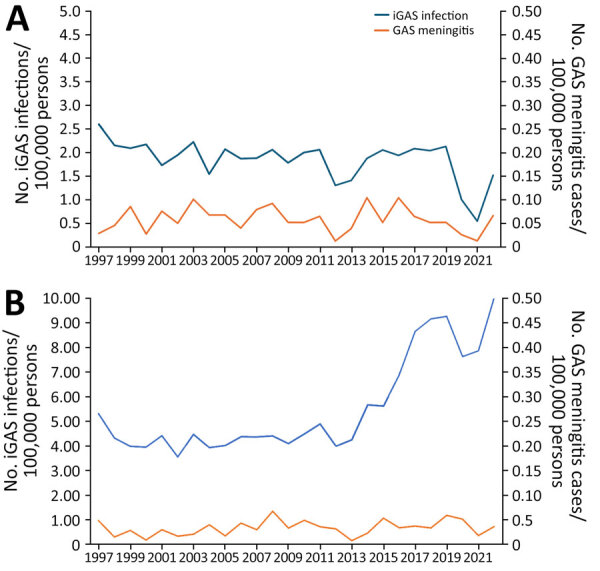
Incidence of iGAS meningitis versus GAS infections, by age, United States, 1997–2022. A) Patients <18 years of age; B) patients >18 years of age. Scales for the y-axes differ substantially to underscore patterns but do not permit direct comparisons. GAS, group A *Streptococcus*; iGAS, invasive GAS.

GAS meningitis incidence was higher in younger age groups, representing 3.2% of all pediatric iGAS cases and with an annual average incidence of 0.06 cases/100,000 children. GAS meningitis represented 0.6% of adult iGAS cases and had an average annual incidence of 0.03 cases/100,000 adults (Figure 1). Children <1 year of age had the highest GAS meningitis incidence, an average annual incidence of 0.23 cases/100,000 children, representing 4.8% of iGAS cases in patients <1 year of age. Adults 18–64 years of age had the lowest GAS meningitis incidence, an average annual incidence of 0.03 cases/100,000 persons. Extrapolating those incidences to the entire country, we estimated 3,528 cases of meningitis caused by GAS in the United States during the 26-year period of 1997–2022, ranging from 49 to 239 cases annually, including 686 fatal cases.

### GAS Meningitis Incidence Compared with Other Bacterial Meningitis Etiologies

During 1997–2022, ABCs received 11,026 reported cases of bacterial meningitis caused by GAS, GBS, *S. pneumoniae*, *N. meningitidis*, or *H. influenzae*. The percentage of meningitis cases caused by GAS increased over time (p<0.001) (Appendix Figure 3).

The average annual incidence of GAS meningitis across all ages (0.04 cases/100,000 persons) was lower than that of meningitis caused by the other pathogens in the 1997–2005 and 2006–2015 periods (Table 3) but similar to the incidence of meningococcal meningitis (0.04 cases/100,000 persons) in 2016–2022. However, the overall CFR of GAS meningitis for each age group was higher (13.1%–26.1%) than that of the other pathogens, except for GBS in the >65 years of age group (Table 3). When stratified by age group (Appendix Figure 4), the percentage of GAS meningitis among all meningitis cases in ABCs was highest (8.7%) in the 5–17 years of age group and lowest (1.1%) in the 0–11 months of age group.

**Table 3:** Incidence and case-fatality rates for GAS meningitis and meningitis caused by other bacterial pathogens, United States, 1997–2022*

Rates per age group	GAS	GBS	*Streptococcus pneumoniae*	*Hemophilus influenzae*	*Neisseria meningitides*
Incidence rate†					
0–11 mo					
1997–2005	0.34	12.30	8.95	1.71	3.04
2006–2015	0.22	12.80	4.57	2.14	1.59
2016–2022	0.15	12.64	2.75	2.71	0.48
All years	0.23	12.61	5.37	2.17	1.71
1–4 y					
1997–2005	0.04	0.03	1.49	0.21	0.73
2006–2015	0.06	0.02	0.61	0.35	0.24
2016–2022	0.07	0.01	0.53	0.35	0.04
All years	0.06	0.02	0.84	0.31	0.32
5–17 y					
1997–2005	0.05	0.03	0.35	0.05	0.45
2006–2015	0.05	0.02	0.25	0.03	0.07
2016–2022	0.04	0.01	0.27	0.05	0.02
All years	0.05	0.02	0.28	0.04	0.16
18–64 y					
1997–2005	0.02	0.09	0.84	0.07	0.23
2006–2015	0.03	0.07	0.70	0.07	0.09
2016–2022	0.03	0.07	0.53	0.08	0.03
All years	0.03	0.07	0.68	0.07	0.11
>65 y					
1997–2005	0.04	0.15	1.50	0.17	0.10
2006–2015	0.06	0.10	1.27	0.16	0.08
2016–2022	0.06	0.17	0.90	0.22	0.04
All years	0.06	0.14	1.18	0.19	0.07
All ages					
1997–2005	0.04	0.26	0.98	0.11	0.32
2006–2015	0.04	0.24	0.74	0.12	0.11
2016–2022	0.04	0.22	0.57	0.14	0.04
All years	0.04	0.24	0.75	0.12	0.14
Case fatality rate, %‡					
0–11 mos	21.7	7.0	6.7	4.7	5.4
1–4 y	26.1	12.5	11.2	2.5	5.5
5–17 y	13.1	4.8	8.1	1.9	10.7
18–64 y	20.9	14.4	15.6	3.8	11.0
>65 y	20.4	26.3	21.3	7.7	15.4
All ages	17.9	9.9	15.2	4.9	9.6

*GAS, group A *Streptococcus*; GBS, group B *Streptococcus*. †Cases per 100,000 population. ‡Rate for 1997–2022.

### *emm* Types and Vaccine Targets

Among GAS meningitis cases, 263 (82.2%) had isolates available for *emm* typing; 153 (58.0%) were cultured from cerebrospinal fluid, 106 (40.1%) from blood, and 5 (1.9%) from other sources. We identified 41 *emm* types among GAS meningitis isolates; the 10 most common *emm* types covered 76.0% of isolates. Overall, nonmeningitis isolates had higher *emm* diversity than meningitis isolates; 136 different *emm* types were identified, and 64.6% of isolates were covered by the 10 most common *emm* types (Appendix Figure 5).

The 2 most common *emm* types among meningitis cases were *emm*1 (31.9%) and *emm*12 (12.2%), accounting for 48.9% of isolates from children and 41.4% from adults. *emm*6 (9.6%) was the third most common type among children and *emm*28 (5.9%) was the third most common among adults. CFR among case-patients with an *emm*1 isolate (26.2%) was significantly higher than CFR among case-patients with other *emm* types (13.3%) (p<0.05). Of the 84 *emm*1 GAS meningitis isolates, 2 isolates identified in 2020 belonged to the M1_UK_ lineage. Among nonmeningitis cases, the most common types were *emm*1 (16.5%) and *emm*89 (7.5%).

**Figure 2:**
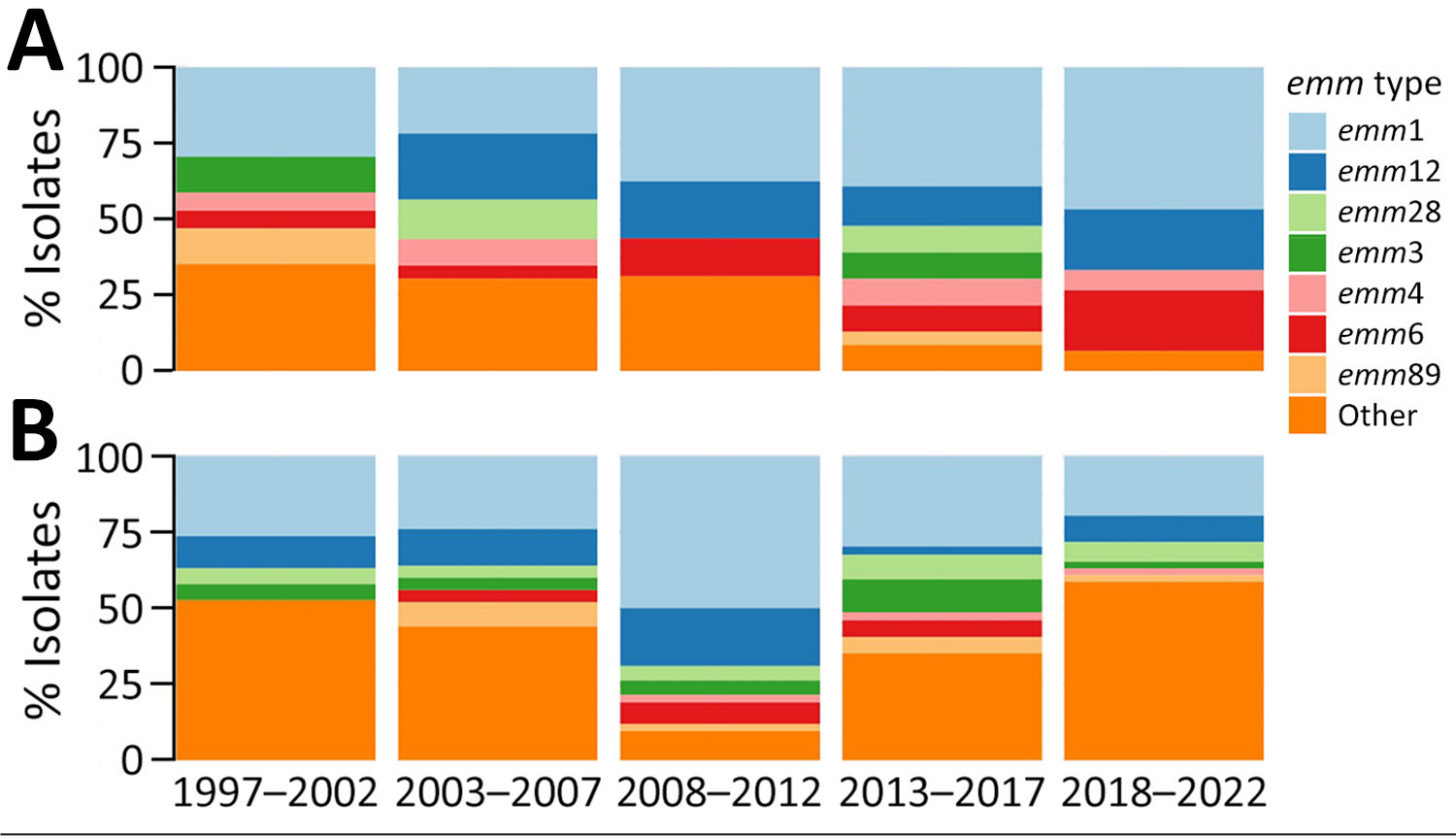
Distribution of *emm* types among isolates from group A *Streptococcus* meningitis cases, by age, United States, 1997–2022. A) Patients <18 years of age; B) patients >18 years of age.

Among children with GAS meningitis, the percentage of *emm*1 isolates ranged from 21.7% during 2008–2012 to 46.7% during 2018–2022; the percentage of isolates belonging to the 7 most common *emm* types (1, 3, 4, 6, 12, 28, and 89) increased from 37.5% (6/16) during 1997–2002 to 93.5% (14/15) during 2018–2022 (Figure 2). Among meningitis isolates from adult patients, the percentage of isolates belonging to the 7 most common *emm* types ranged from 41.3% (19/46) during 2018–2022 to 90.5% (38/42) during 2008–2012. The *emm* types included in the 30-valent vaccine candidate represented 91.5% of isolates from pediatric meningitis cases and 89.9% of isolates from adult meningitis cases.

### Antimicrobial Resistance

**Figure 3:**
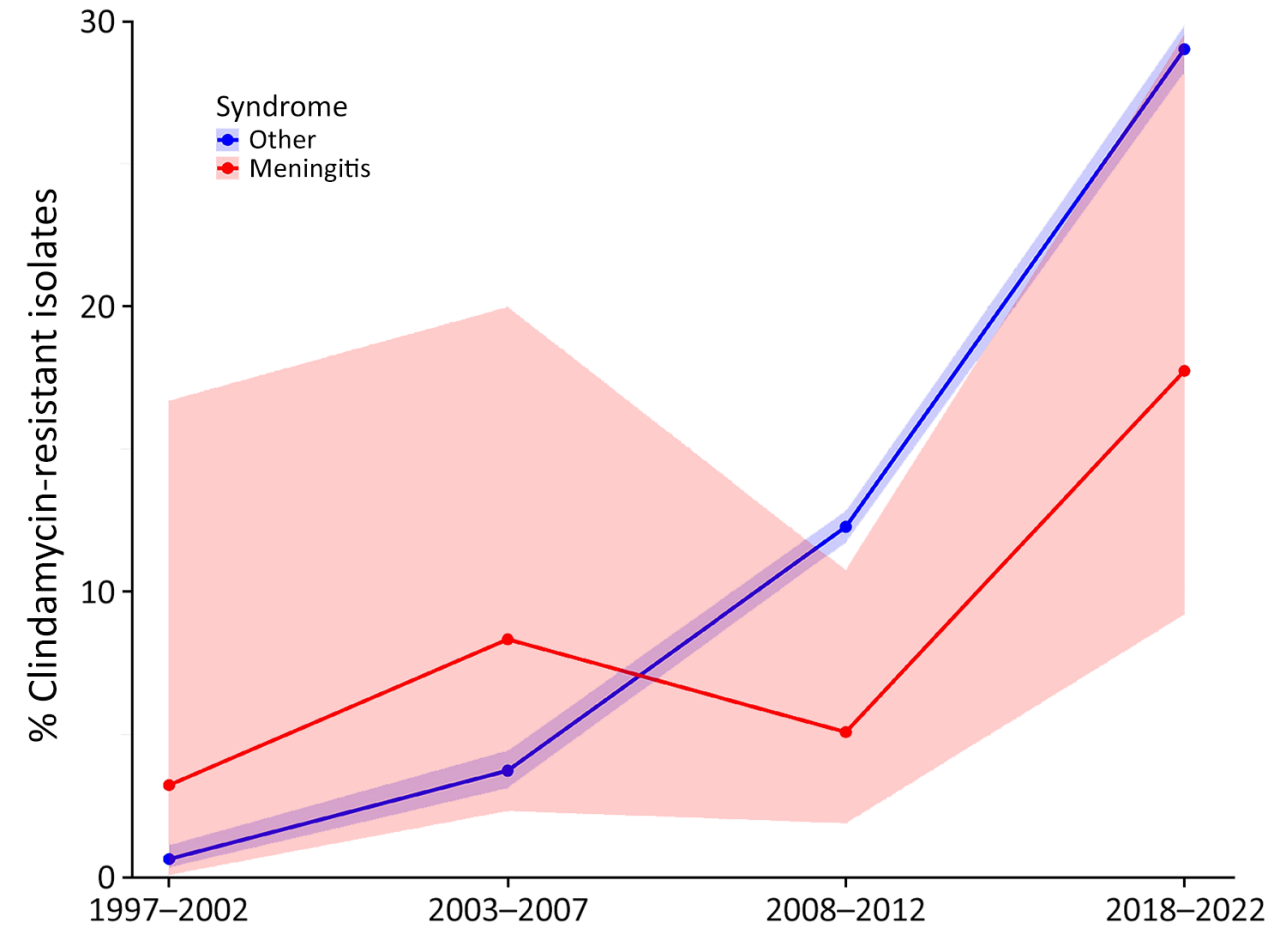
Clindamycin resistance among isolates from group A *Streptococcus* (GAS) meningitis and other GAS-related syndromes, United States, 1997–2022. The graph demonstrates increasing rates of clindamycin resistance among GAS isolates over time. Shading indicates 95% CIs.

Among meningitis cases, 259 (80.9%) had isolates available for antimicrobial drug susceptibility testing. We determined or predicted 25 (10.0%) isolates were resistant to erythromycin, of which 21 (84.0%) were also resistant to clindamycin, 1 (4.0%) was also resistant to levofloxacin, and 1 (4.0%) was also resistant to chloramphenicol. We did not detect β-lactam, linezolid, or vancomycin resistance among available isolates. Of the 21 clindamycin-resistant isolates, 8 (38.1%) were *emm*92 and 4 (19.1%) were *emm*12 type; all *emm*92 isolates were part of the same ST82 lineage. The percentage of clindamycin-resistant isolates among GAS meningitis cases increased over time (Figure 3), from 3.2% (n = 1) during 1997–2002 to 17.7% (n = 11) during 2018–2022 (p<0.05), but remained lower than rates among nonmeningitis cases, from which clindamycin resistance increased from 0.6% (n = 10) during 1997–2002 to 29.0% (n = 3,088) during 2018–2022 (p<0.05).

## Discussion

We explored trends in GAS meningitis in the United States over 26 years and demonstrated that meningeal infection is an uncommon but severe manifestation of iGAS and that GAS meningitis incidence is similar to that of more widely recognized meningitis etiologies. Although the incidence of bacterial meningitis in the United States has decreased overall since 2008 (*10*), driven largely by decreases in *S. pneumoniae* and *N. meningitidis*, the incidence of GAS meningitis remained stable across the years of the study. However, the percentage of bacterial meningitis caused by GAS in the United States has substantially increased over time. Starting in 2022, multiple countries reported increases in iGAS disease and meningitis (*2*,*8*,*9*), suggesting that GAS could further expand as a meningitis etiology. Although the overall incidence of GAS meningitis remains lower than those for meningitis caused by *S. pneumoniae, H. influenzae*, and GBS, the CFR was 1.5–4 times higher for GAS meningitis. In this large cohort, the 19.4% CFR for GAS meningitis closely approximates that seen in prior studies (*16*,*17*), and CFR did not decrease over time.

In our study, children <1 year of age had the highest incidence of GAS meningitis and the second highest CFR, consistent with previous reports of GAS meningitis in young children (*18*–*20*). Preliminary ABCs data for 2023 (P.A. Hawkins et al., unpub. data) showed the incidence of GAS meningitis in that age group increased from 0.15 (95% CI 0.04–0.37) cases/100,000 children during 2016–2022 to 1.06 (95% CI 0.26–2.38) cases/100,000 children, even though the overall incidence of GAS meningitis remained stable. Compared with other etiologies of bacterial meningitis in ABCs among children <1 year of age, GAS still represents a small percentage of meningitis cases, most of which are caused by GBS.

We observed a seasonal pattern of GAS meningitis cases, and a peak in winter and early spring; that same pattern has been reported for GAS pharyngitis and iGAS infections in the United States and Europe (*1*,*5*,*6*,*14*). One study proposed that the seasonal pattern might be explained by concurrently circulating viral infections during high-incidence periods, along with effects of crowding and close contact because of increased time spent indoors during cold months (*1*).

We also noted that the percentage of GAS meningitis patients with underlying conditions was low, particularly among pediatric patients, and that >90% of cases occurring in children without underlying medical conditions, potentially reflecting the severity of *emm*1 infections, as previously documented (*1*). Although most GAS meningitis cases in our study occurred in previously healthy children, nearly 1 in 5 pediatric GAS meningitis cases were fatal. We noted otitis media in ABCs for 13.8% of all GAS meningitis cases, consistent with previous reports that found that GAS meningitis was often preceded or accompanied by otitis media or sinusitis (*16*,*17*,*20*–*23*) and likely resulted from continuous spread of the infection rather than particular virulence factors (*24*)

The 2 most common *emm* types we identified among GAS meningitis isolates, *emm*1 and *emm*12, belonged to the *emm* pattern A-C, which has a strong preference for infection at the throat (*25*,*26*). *emm*1 and *emm*12 are among the most common *emm* types in pharyngitis isolates reported across the United States, Canada, and Europe (*15*,*27*–*30*). *emm*1 has also been documented as the predominant strain from GAS meningitis cases in small studies from several countries in Europe, which identified increases in overall iGAS and GAS meningitis incidence associated with *emm*1, particularly the M1_UK_ lineage (*1*,*8*,*9*,*16*,*17*,*20*,*31*–*33*). However, despite a substantial increase in M1_UK_ isolates in ABCs sites in the United States from 2015–2018 to 2019–2021 (*34*), only 2 of 263 meningitis isolates typed in our study belonged to the M1_UK_ lineage. Isolates from GAS meningitis cases in this study were less diverse overall than those from other iGAS infections, highlighting the potential for vaccines in development to provide substantial protection against this disease manifestation.

The first-line antimicrobial regimen used in the empiric treatment of bacterial meningitis is typically a combination of vancomycin plus a third-generation cephalosporin, or a fluoroquinolone for patients who might have a severe allergy to β-lactams (*35*). Clindamycin combined with β-lactams has been associated with improved survival in severe iGAS infections (*36*) and is frequently used as part of combination antimicrobial therapy in intracranial GAS infections (*21*,*22*). We did not observe any β-lactam or vancomycin resistance among the 259 isolates tested and found only 1 isolate resistant to fluoroquinolones. On the other hand, clindamycin resistance among GAS meningitis isolates substantially increased across the years of this study, consistent with a reported 3-fold increase in macrolide and clindamycin resistance from 2013 to 2022 among iGAS isolates in ABCs (*2*). The poor diffusion of clindamycin over the meningeal membranes is an additional challenge and has led to recommendations to use high-dose clindamycin or linezolid as an adjunctive antibiotic (*37*,*38*).

One limitation of our study was the low incidence of GAS meningitis reported in ABCs, which made it difficult to detect patterns from year to year; however, we were able to show trends in the epidemiology of GAS meningitis in the United States over time. A limitation posed by ABCs data is the focus on invasive disease, which does not fully capture concurrent noninvasive GAS disease syndromes such as otitis media and pharyngitis; those syndromes have been reported in a high percentage of GAS meningitis cases in previous studies. Another limitation of ABCs is that it does not collect outcome data after discharge, which is necessary to assess long-term sequelae and delayed death. Neurologic sequelae occur in a large percentage of patients who survive GAS meningitis (*7*,*16*,*17*). Finally, we did not collect antibiotic use history and thus were unable to determine any correlation between antibiotic use and antimicrobial resistance or outcome in GAS meningitis cases.

In summary, we assessed 26 years of clinical, epidemiologic, and molecular data available from the well-established and robust ABCs system, which provided a large collection of GAS meningitis cases. Our findings provide insights into GAS meningitis, its epidemiology and clinical manifestations, and its contribution to the overall burden of bacterial meningitis in the United States since evolution of bacterial meningitis epidemiology after introduction and sustained use of vaccines against other pathogens causing meningitis, including *S. pneumoniae*, *N. meningitidis*, and *H. influenzae*. Our findings highlight the need for including GAS in future meningitis studies and reviews and in updated meningitis clinical practice guidelines and can help inform clinical practice, vaccine design, and public health interventions in the United States. Clinicians should be aware that meningitis is an uncommon but severe manifestation of invasive GAS and has a higher CFR than other meningitis etiologies and, therefore, should consider GAS in the differential diagnosis of meningitis.

Appendix Additional information on group A *Streptococcus* meningitis, United States, 1997–2022.
