# Supplementary material for: Group A Streptococcus Meningitis, United States, 1997–2022
Source: Emerg Infect Dis. 2026 Jan;32(1):34–44. doi: 10.3201/eid3201.250871 (PMC12870060; doi:10.3201/eid3201.250871)
Supplement: Appendix — Additional information on group A Streptococcus meningitis, United States, 1997–2022. [file 25-0871-Techapp-s1.pdf]

Article DOI: https://doi.org/10.3201/eid3201.250871

EID cannot ensure accessibility for supplementary materials supplied by authors. Readers who have difficulty accessing supplementary content should contact the authors for assistance.

Group A *Streptococcus* Meningitis, United States, 1997–2022

Appendix

Appendix Table. ABCs Surveillance Population Matrix, 1997–2022

Group A strep	CA	CO	CT	GA	MD	MN	NM	NY	OR	TN
1997	San Francisco, Contra Costa, Alameda counties		Entire state	Barrow, Bartow, Carroll, Cherokee, Clayton, Cobb, Coweta Dekalb, Douglas, Fayette, Forsyth, Fulton, Gwinnett, Henry, Newton, Paulding, Pickens, Rockdale, Spalding, Walton counties	Anne Arundel, Baltimore, Baltimore City, Carroll, Harford, and Howard counties	Anoka, Carver, Dakota, Hennepin, Ramsey, Scott, Washington counties			Clackamas, Multnomah, Washington counties	
1998	San Francisco, Contra Costa, Alameda counties		Entire state	Barrow, Bartow, Carroll, Cherokee, Clayton, Cobb, Coweta Dekalb, Douglas, Fayette, Forsyth, Fulton, Gwinnett, Henry, Newton, Paulding, Pickens, Rockdale, Spalding, Walton counties	Anne Arundel, Baltimore, Baltimore City, Carroll, Harford, and Howard counties	Anoka, Carver, Dakota, Hennepin, Ramsey, Scott, Washington counties		Genesee, Livingston, Monroe, Ontario, Orleans, Wayne, Yates counties	Clackamas, Multnomah, Washington counties	
1999	San Francisco, Contra Costa, Alameda counties		Entire state	Barrow, Bartow, Carroll, Cherokee, Clayton, Cobb, Coweta Dekalb, Douglas, Fayette, Forsyth, Fulton, Gwinnett, Henry, Newton, Paulding, Pickens,	Anne Arundel, Baltimore, Baltimore City, Carroll, Harford, and Howard counties	Entire state		Albany, Columbia, Genesee, Greene, Livingston, Monroe, Montgomery, Ontario,	Clackamas, Multnomah, Washington counties	Davidson, Hamilton, Knox, Shelby, Williamson counties

Group A strep	CA	CO	CT	GA	MD	MN	NM	NY	OR	TN
				Rockdale, Spalding, Walton counties				Orleans, Rensselaer, Saratoga, Schenectady, Schoharie, Wayne, Yates counties		
2000	San Francisco, Contra Costa, Alameda counties		Entire state	Entire state	Anne Arundel, Baltimore, Baltimore City, Carroll, Harford, and Howard counties	Entire state		Albany, Columbia, Genesee, Greene, Livingston, Monroe, Montgomery, Ontario, Orleans, Rensselaer, Saratoga, Schenectady, Schoharie, Wayne, Yates	Clackamas, Multnomah, Washington counties	Cheatham, Davidson, Dickson, Hamilton, Knox, Robertson, Rutherford, Shelby, Sumner, Williamson, and Wilson
2001–2003	San Francisco, Contra Costa, Alameda counties	Adams, Arapahoe, Denver, Douglas, and Jefferson counties	Entire state	Entire state	Anne Arundel, Baltimore, Baltimore City, Carroll, Harford, and Howard counties	Entire state		Albany, Columbia, Genesee, Greene, Livingston, Monroe, Montgomery, Ontario, Orleans, Rensselaer, Saratoga, Schenectady, Schoharie, Wayne, Yates	Clackamas, Multnomah, Washington counties	Cheatham, Davidson, Dickson, Hamilton, Knox, Robertson, Rutherford, Shelby, Sumner, Williamson, and Wilson counties
2004–2009	San Francisco, Contra Costa, Alameda counties	Adams, Arapahoe, Denver, Douglas, and Jefferson counties	Entire state	Barrow, Bartow, Carroll, Cherokee, Clayton, Cobb, Coweta Dekalb, Douglas, Fayette, Forsyth, Fulton, Gwinnett, Henry, Newton, Paulding, Pickens, Rockdale, Spalding, Walton counties	Anne Arundel, Baltimore, Baltimore City, Carroll, Harford, and Howard counties	Entire state	Entire state	Albany, Columbia, Genesee, Greene, Livingston, Monroe, Montgomery, Ontario, Orleans, Rensselaer, Saratoga, Schenectady, Schoharie, Wayne, Yates	Clackamas, Multnomah, Washington counties	Cheatham, Davidson, Dickson, Hamilton, Knox, Robertson, Rutherford, Shelby, Sumner, Williamson, and Wilson counties

Group A strep	CA	CO	CT	GA	MD	MN	NM	NY	OR	TN
2010–2022	San Francisco, Contra Costa, Alameda counties	Adams, Arapahoe, Denver, Douglas, and Jefferson counties	Entire state	Barrow, Bartow, Carroll, Cherokee, Clayton, Cobb, Coweta Dekalb, Douglas, Fayette, Forsyth, Fulton, Gwinnett, Henry, Newton, Paulding, Pickens, Rockdale, Spalding, Walton counties	Anne Arundel, Baltimore, Baltimore City, Carroll, Harford, and Howard counties	Entire state	Entire state	Albany, Columbia, Genesee, Greene, Livingston, Monroe, Montgomery, Ontario, Orleans, Rensselaer, Saratoga, Schenectady, Schoharie, Wayne, Yates	Clackamas, Multnomah, Washington counties	Anderson, Blount, Cheatham, Davidson, Dickson, Grainger, Hamilton, Jefferson, Knox, Loudon, Madison, Roane, Robertson, Rutherford, Sevier, Shelby, Sumner, Union, Williamson, Wilson counties

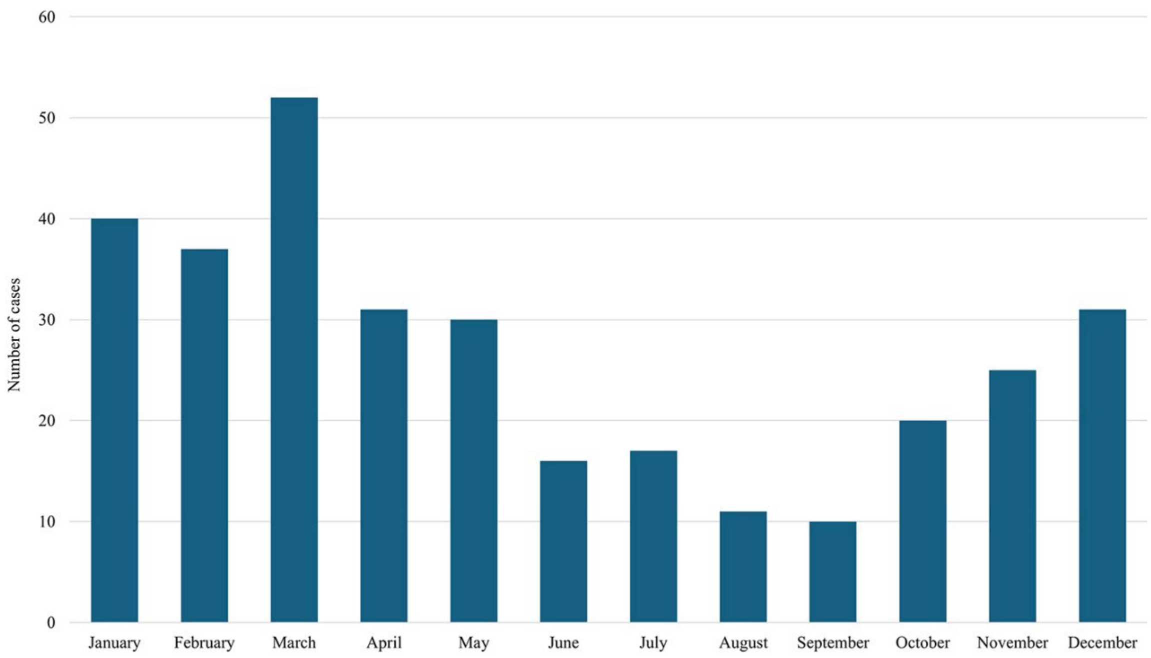

Appendix Figure 1. Number of meningitis cases identified per month, 1997–2022.

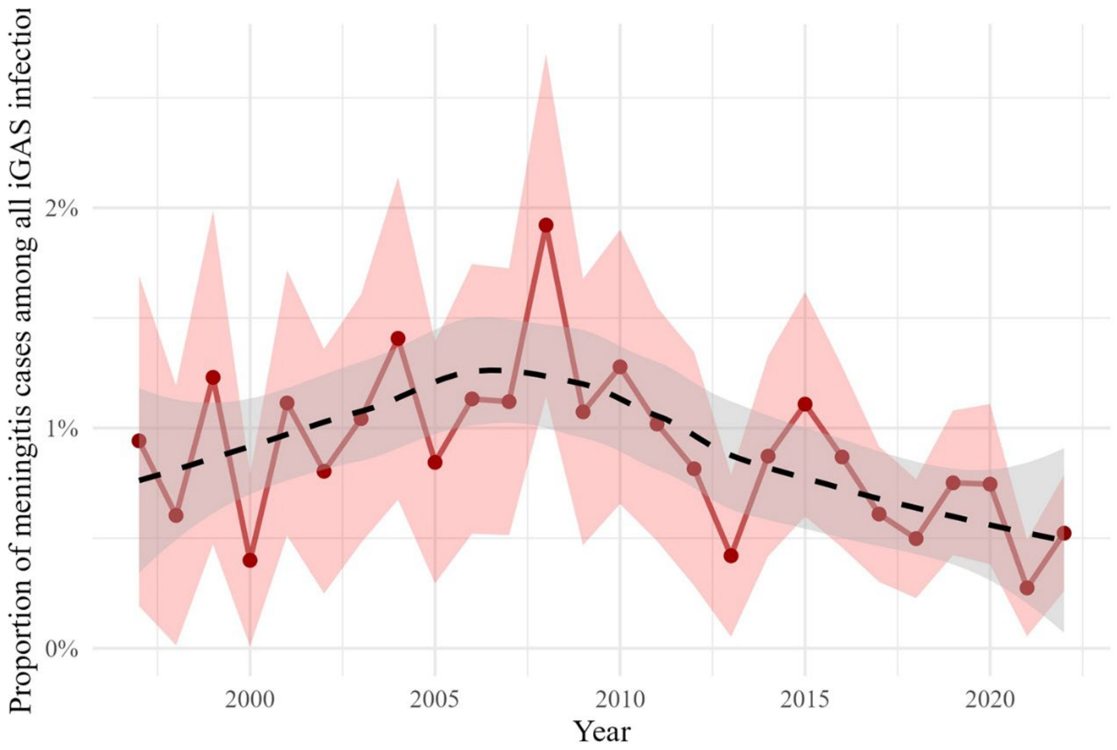

Appendix Figure 2. Percent of meningitis cases among invasive GAS infections, 1997–2022.

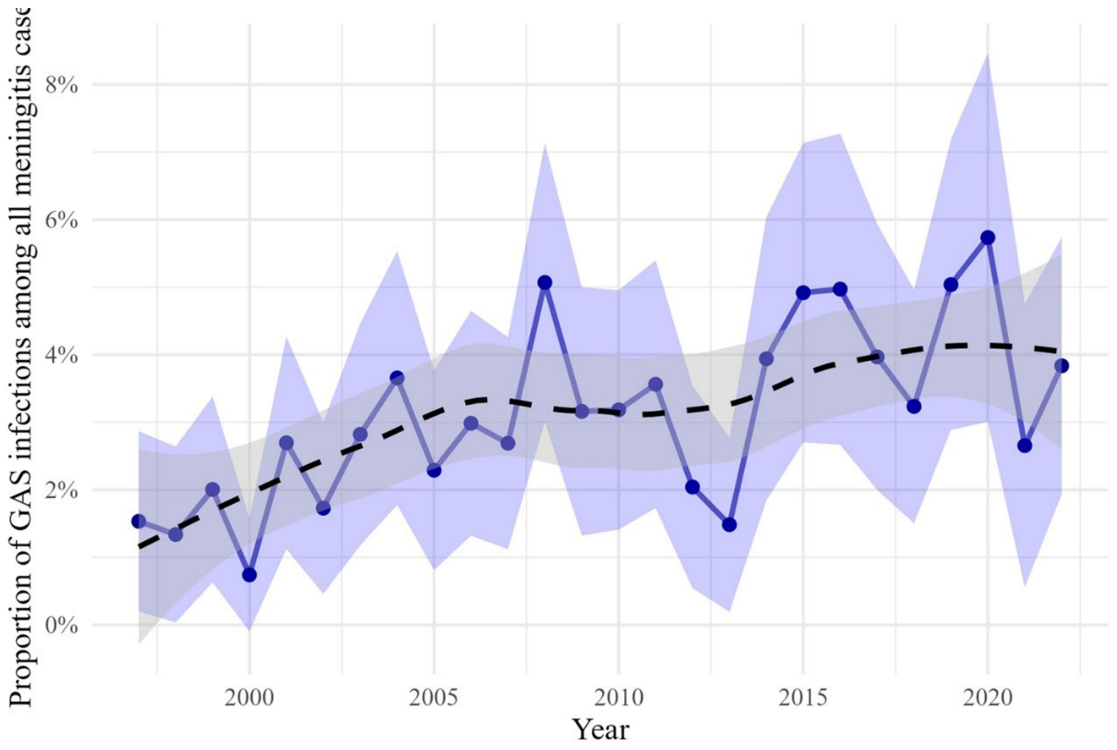

Appendix Figure 3. Percent of GAS infections among all meningitis cases in ABCs, 1997–2022.

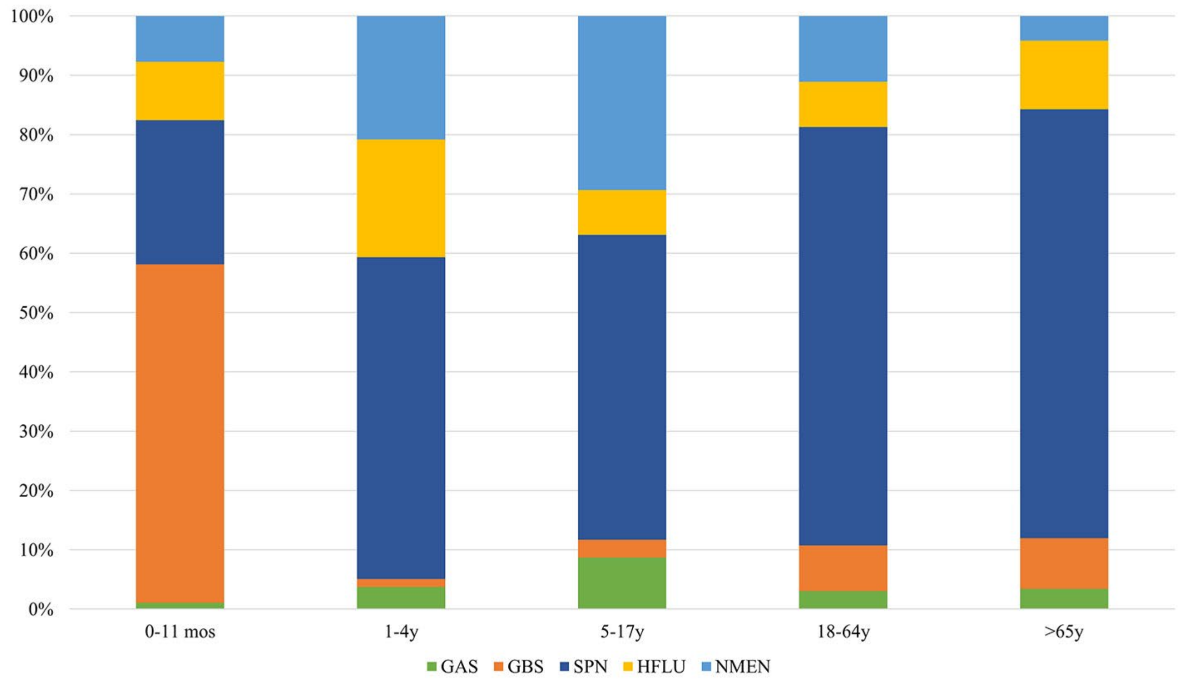

Appendix Figure 4. Etiologic fraction of meningitis causing pathogens in ABCs by age group.

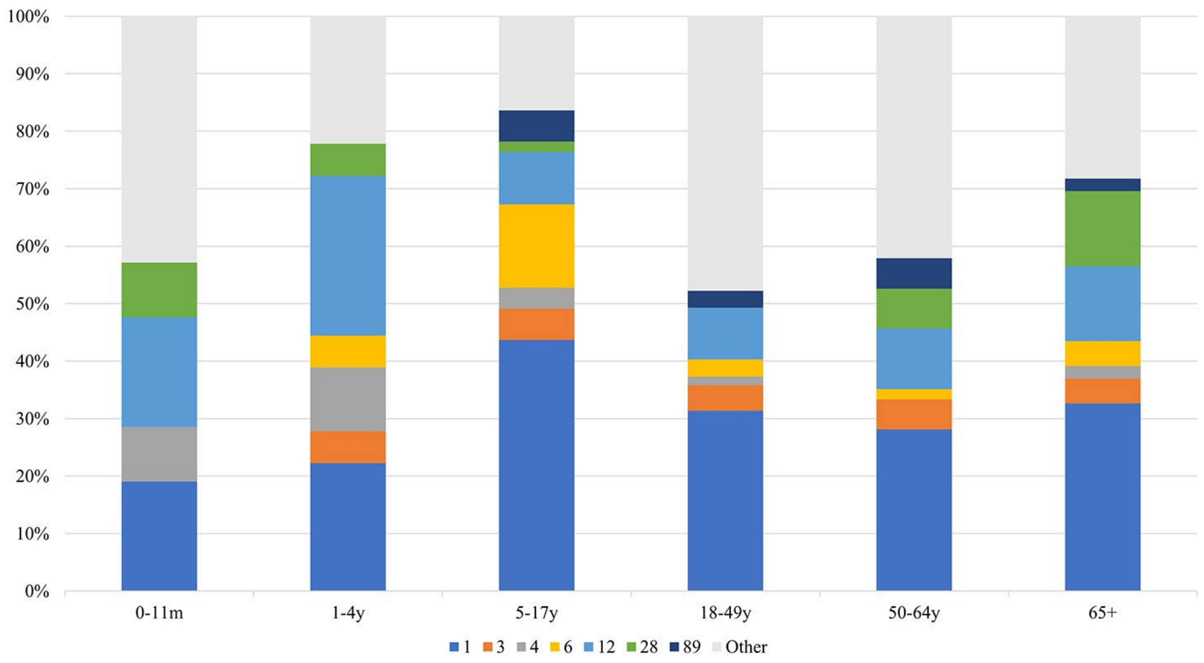

Appendix Figure 5. *Emm* type distribution among GAS meningitis isolates, by age group.
